# Internal jugular vein catheterization and catheter insertion slipped into the cervical spinal canal: a special case report

**DOI:** 10.3389/fmed.2026.1845016

**Published:** 2026-06-16

**Authors:** Jinshuai Zhai, Teng Huang, Xiao Zheng, Bo Gao, Long Zhang, Wenyi Li

**Affiliations:** The Department of Orthopedics, Hebei General Hospital, Shijiazhuang, Hebei, China

**Keywords:** central venous catheterization, cervical spine, internal jugular vein, puncture, spinal cord

## Abstract

This paper reports a case of accidental entry of an internal jugular vein catheter into the cervical spinal canal. A male patient in his fifties sustained injuries in a road traffic accident. During the placement of a catheter into the right internal jugular vein, the catheter inadvertently entered the cervical spinal canal, though no damage was caused to the spinal cord or nerves. A second successful puncture and catheter placement was performed under ultrasound guidance, and the patient eventually made a full recovery and was discharged. This case is extremely rare and of particular clinical significance, highlighting the importance of monitoring during the insertion and removal of catheters for internal jugular vein puncture.

## Introduction

Internal jugular vein catheterization serves as a fundamental approach for central venous access, enabling reliable infusion of fluids, medication administration, and hemodialysis (1). The right internal jugular vein is the preferred site due to its direct trajectory toward the superior vena cava, contributing to higher technical success rates and lower complication frequencies compared to alternative routes. Nevertheless, procedural adverse events—including hematoma formation, arterial puncture, pneumothorax, and arrhythmia—may arise from operator inexperience or vascular anatomical variations (2–4). Notably, Inadvertent penetration into the cervical spinal canal during internal jugular vein catheterization is an exceedingly rare complication, and the preservation of intact spinal cord function in such incidents is exceptionally uncommon. This case report details an incident of cervical spinal canal misplacement during right Internal jugular vein catheterization at Hebei General Hospital, analyzing causative factors and proposing preventive strategies to mitigate such occurrences.

## Case presentation

The patient, approximately a 50-year-old male, was admitted to the hospital mainly due to a “injury for 3 h”. On admission, the patient’s Glasgow Coma Scale score was 10. CT revealed splenic rupture, abdominal effusion, multiple rib fractures, lung contusion, and pelvic fracture. Vital signs: body temperature: 36.7 °C, blood pressure: 75/52 mmHg, heart rate: 111 bpm, respiratory rate: 35 breaths/min, oxygen saturation (SpO_2_): 98%. On admission, coagulation tests were performed: PT: 14.8 s, APTT: 23 s, TT: 16.2 s, INR: 0.88, D-dimer: 80 mg/L. In order to maintain stable vital signs, the central venous fluid route was established by an attending physician in the emergency department. The patient was placed supine with the head tilted to the left. The upper border of the sternal head of the right sternocleidomastoid muscle was used as the center point of the triangle. After routine disinfection and placement of a sterile drape, the puncture needle was inserted at a 30° angle to the skin. Dark red blood was aspirated when the needle was advanced approximately 3.0 cm from the puncture site. Following guidewire insertion, a central venous catheter (7 Fr-20 cm, double lumen) was successfully inserted to a depth of 13 cm. The catheter insertion process was smooth without any obstruction, and fluid infusion was smooth. The patient had two peripheral IV lines in place, ensuring an adequate supply of intravenous fluids. Considering the damaged spleen and abdominal blood accumulation, he was directly admitted to the operating room for emergency surgery. During the multidisciplinary consultation, the orthopedic surgeon reviewed the imaging and identified an abnormality in the cervical spinal canal, specifically a hyperdense shadow within the canal. The differential diagnosis included other possibilities, such as a spinal cord stimulator lead; however, the patient was previously healthy with no history of surgery. In light of the central venous catheterization procedure, it was determined that the central venous catheter had been misplaced into the cervical spinal canal. The puncture catheter penetrated the cervical spinal canal through the right C4–C5 intervertebral foramen (Figures 1a, b) and penetrated the soft tissues of the chest through the right C6–C7 intervertebral foramen (Figures 1a, c). However, due to the patient’s unconsciousness and inability to cooperate with the physical examination, the spinal surgeon was unable to assess the presence of spinal cord or nerve root injury. It was recommended that the central venous catheter be adjusted, so the right catheter was removed and replaced with a left internal jugular central venous catheter under ultrasonographic guidance by Associate Chief Physician, Department of Anesthesiology (155 min after the initial catheterization). During the removal process, ultrasound monitoring was used, the catheter was slowly withdrawn, and bedside C-arm fluoroscopy was employed to confirm complete removal. Because the patient was unconscious, the presence of spinal cord or nerve injury could not be definitively determined. On postoperative day 3, after the patient regained consciousness, physical examination revealed no signs of neurological deficits. Neurological function remained intact until the day of discharge, and no complications such as hematoma or infection occurred during hospitalization. Telephone follow-ups conducted at 4 and 6 weeks after discharge revealed no delayed neurological, bleeding, infectious, or vascular complications. Subsequent chest computed tomography (Figure 2) revealed that the catheter was in good position. After the procedure, the patient was transferred to the intensive care unit (ICU) for further management. Once the patient’s condition stabilized, he was transferred to the general ward and eventually discharged after recovery. The timeline of each event is shown in Figure 3.

**FIGURE 1:**
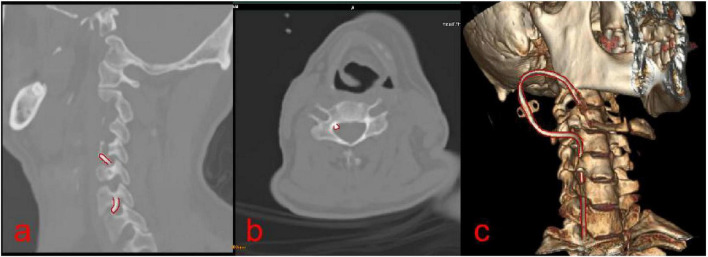
**(a)** Sagittal CT scan of the cervical spine shows a catheter entering the cervical spinal canal through the right C4–C5 intervertebral foramen and exiting through the right C6–C7 intervertebral foramen. **(b)** Axial CT scan of the cervical spine demonstrates a round hyperdensity within the spinal canal at the C5 vertebral level. **(c)** Three-dimensional CT reconstruction of the cervical spine.

**FIGURE 2:**
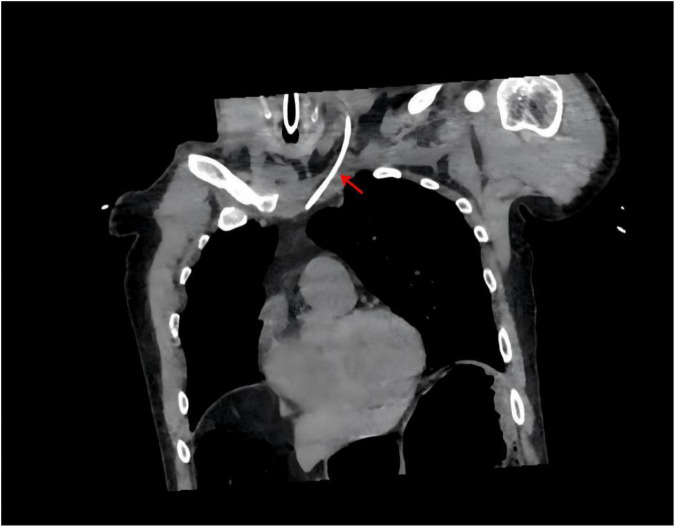
Chest CT shows the replacement catheter following repositioning via the contralateral left internal jugular vein.

**FIGURE 3:**
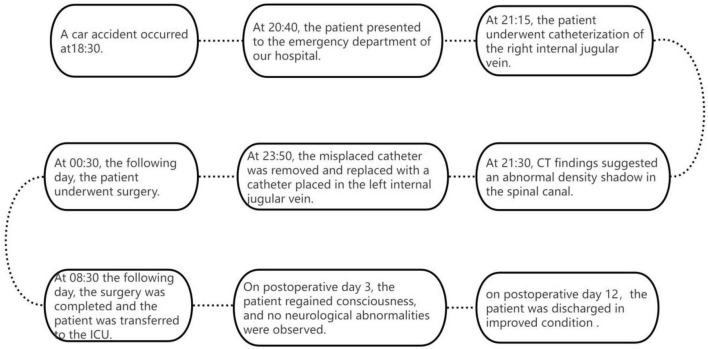
Timeline of each event.

## Discussion

Central venous catheterization is an essential skill of intensivists, among which internal jugular venipuncture catheterization is the most commonly used catheterization approach, which can be used for fluid rehydration, measurement of central venous pressure, parenteral nutrition, drug application, and other treatments (5). It has the advantages of long retention time and low incidence of phlebitis. Due to its special anatomical relationship, internal jugular vein catheterization is relatively simple, fast, and has a high success rate. However, there are still certain risks, such as artery injury, puncture failure, hematoma, nerve injury, pneumothorax, and other (3). In our case report, although an abnormal catheter position was observed, common complications such as pneumothorax or nerve injury did not occur; instead, a rarer scenario emerged—the catheter entered the spinal canal via an intervertebral foramen and subsequently exited through another intervertebral foramen, without presenting any symptoms suggestive of spinal cord injury. To the best of our knowledge, this appears to be an exceptionally rare case of internal jugular venous catheter misplacement into the spinal canal. The uniqueness of this case lies in the exceptionally complex trajectory of the catheter. Typically, accidental entry into the spinal canal during internal jugular vein puncture is caused by the needle penetrating too deeply or at an incorrect angle, directly piercing the vertebral body or intervertebral foramen to enter the spinal canal. However, in this case, the catheter not only entered the spinal canal via the right C4-5 intervertebral foramen but also continued its descent, emerging from the right C6-7 intervertebral foramen into the pleural soft tissue, forming a “trans-spinal canal” pathway traversing the cervical spinal canal.

Ultrasound-guided internal jugular vein puncture and catheterization, owing to its non-invasive nature and visual guidance, can significantly improve the success rate of the procedure, reduce the risk of complications, and thereby effectively minimize iatrogenic injury (6). Studies have shown that beginners need to complete at least 14 operations to master the ultrasound-guided internal jugular vein catheterization technique (7), while for units without ultrasound guidance, experienced and solidly anatomically qualified doctors may be required to reduce risk and damage as much as possible.

We conducted a literature search in the CNKI, Wanfang, and PubMed databases for studies on complications associated with central venous catheterization. Several authors have reported relevant cases: Ma et al. (8), Kohyama et al. (9), Gong et al. (10), and colleagues described instances of catheter misdirection into the cephalic vein, right subclavian artery, or thoracic cavity during ultrasound-guided right internal jugular vein catheterization. Ghoddusi et al. (11) reported a case of catheter misplacement into the posterior mediastinum during right internal jugular vein puncture performed without ultrasound guidance. Although all these cases led to corresponding symptoms, none resulted in adverse outcomes. Our case differs significantly from previously reported cases. In previous reports, catheters inadvertently placed into the spinal canal often remained lodged within the canal, and most patients developed immediate neurological dysfunction due to spinal cord compression or injury, requiring emergency surgical removal of the catheter. In contrast, in our case, the entire course of the catheter did not cause substantial compression or injury to the spinal cord, and its trajectory was unusual. The catheter was ultimately successfully and safely removed percutaneously under ultrasound guidance, with no residual neurological deficits after surgery. Our case further expands the clinical spectrum of this rare complication.

In this case, based on CT examination, we inferred that the catheter entered the cervical spinal canal through the right intervertebral foramen at the C4–C5 level and exited through the right intervertebral foramen at the C6–C7 level. The upper and lower walls of the intervertebral foramen were the adjacent pedicle notch, the anterior wall was the posterolateral vertebral body and intervertebral disk, and the posterior wall was the facet joint and ligamentum flavum. The loose connective tissue area outside the dural sheath of the nerve root and between the walls of the intervertebral foramen allowed the catheter to move within the epidural space. Fortunately, the catheter did not damage the cervical spinal cord or nerve root. Analysis of its causes may be as follows: Firstly, the patient was in a state of hypovolaemia due to multiple traumas, resulting in a significant reduction in internal jugular vein filling, which greatly increased the difficulty of locating the puncture site; secondly, the patient’s critical condition and the complex on-site situation may have prevented the operator from strictly adhering to standard operating procedures; furthermore, the patient was positioned in the supine position with the head turned to the left, causing the neck muscles to relax and the intervertebral foramen to remain relatively open, thereby facilitating the catheter’s exit. Furthermore, in this case, anatomical landmarks were used for guidance rather than real-time ultrasound guidance. In the absence of adequate vascular filling, reliance solely on superficial anatomical landmarks is significantly less reliable; moreover, no further verification was performed after catheter placement to confirm successful positioning.

Therefore, to prevent complications, physicians who are experienced and possess comprehensive anatomical knowledge may position the patient’s head laterally. The success rate of catheter placement can be effectively increased by selecting an appropriate puncture angle, direction, and depth, in conjunction with ultrasound guidance (12). A study by Khazaei et al. (13) suggests that the success rate of ultrasound-guided transjugular catheterization is significantly higher than that of non-ultrasound-guided catheterization (80.65% vs. 35.48%). A study by Rando et al. (14) showed that the incidence of complications in the ultrasound group was 8.1%, significantly lower than the 14.9% in the non-ultrasound group. The study indicated that ultrasound-guided catheterization is superior to conventional methods in terms of access time, success rate and incidence of complications. A Cochrane systematic review analysing 14 studies (involving a total of 2,406 patients) similarly demonstrated that the use of two-dimensional ultrasound guidance reduces the incidence of complications by 71% and the risk of haematoma formation by 73% (12).

The clinical management of this case fully demonstrates the importance of multidisciplinary collaboration. During the review of imaging studies, the orthopedic surgeon keenly identified an abnormal dense shadow within the cervical spinal canal; by combining this with the patient’s history of catheter placement, this rare complication was promptly recognized, thereby effectively preventing serious consequences. The management approach—involving re-placement of the catheter under ultrasound guidance and confirmation of its correct position—is consistent with current safety principles for central venous access management. Notably, after the central venous catheter was misplaced, the patient did not exhibit any obvious abnormal reactions to drug administration.

Furthermore, the patient presented with preoperative disturbance of consciousness and subsequently underwent surgical treatment under general anesthesia. Consequently, the assessment of neurological function was subject to inherent limitations. The lack of postoperative neurological deficits should be regarded as somewhat fortuitous. This underscores the particular necessity of reconfirmation following catheter placement in trauma patients, particularly those with impaired consciousness or under anesthesia. This single case report lacked control cases, and the trajectory of the malpositioned catheter was partially inferred. Although the initial catheter placement was abnormal, the second placement was adjusted under ultrasound guidance, ultimately achieving smooth infusion. The chest CT (Figure 2) indicated that the puncture catheter was in good position. During hospitalization, the patient did not show any abnormal reactions associated with catheter displacement. After discharge, we carried out a short - term telephone follow - up and found no complications like hematoma or delayed neurological deficits.

However, neither ultrasound guidance nor traditional internal jugular vein catheterization can avoid the occurrence of puncture-related complications. Therefore, following the placement of an internal jugular vein catheter, X-ray or ultrasound can be used to confirm the catheter’s position; where necessary, a CT scan can provide more precise localization and assessment, thereby minimizing complications arising from improper placement.

## Conclusion

Cases of internal jugular vein catheterization inadvertently entering and penetrating the cervical spinal canal are extremely rare, and their occurrence involves multiple factors, including anatomical variations, technical aspects of the procedure, and specific patient conditions. Although the incidence of such complications is extremely low, clinicians must remain highly vigilant. The successful management of this case was attributable to prompt imaging identification, multidisciplinary collaboration, and a standardized re-catheterization procedure, which ultimately averted serious consequences. Clinicians should be fully aware of this potential risk, adopt ultrasound guidance as standard practice, and establish a mechanism for rapid post-placement confirmation to minimize the risk of such rare but serious complications.

## Data Availability

The raw data supporting the conclusions of this article will be made available by the authors, without undue reservation.
